# Tripartite Mentality*The annual address before The American Medico-Psychological Association, at San Antonio, Texas, April, 1905.

**Published:** 1905-07

**Authors:** J. T. Searcy

**Affiliations:** Tuscaloosa, Alabama; Supt. Alabama State Insane Hospital


### TEXAS MEDICAL JOURNAL.

ESTABLISHED JULY, 1885.

PUBLISHED MONTHLY.—SUBSCRIPTION $1.00 A YEAR.

VOL. XXI. AUSTIN, JULY, 1905. No. 1.

For Texas Medical Journal.

### Tripartite Mentality.*

BY J. T. SEARCY, M. D., TUSCALOOSA, ALABAMA.
Supt. Alabama State Insane Hospital.

### MENTALITY.

I prefer to use the word mentality. More readily than the word mind it can be used to designate a property, a qualification, an accomplishment, a faculty, which can be scientifically observed, classified and studied.

Mentality varies normally and, sometimes, abnormally, in the course of the life of the individual; and it very evidently differs in its capabilities and its accomplishments in different individuals.

Mentality is normal when its exhibitions are such as are usual, natural, customary and expected. It is abnormal when it varies from what is usual, natural, customary and expected.

An abnormal exhibition of mentality of any grade is a psychosis.

When the abnormality of any kind of a psychosis reaches such a grade of deficiency or defectiveness as to bring the person within the cognizance and jurisdiction of the law, as the case may be, it is called "craziness," "idiocy," "lunacy," "insanity," and the like. I prefer to confine these terms to such grades as require legal attention. Indeed, the State reserves to itself the right to apply them. A court alone is authorized to declare a person disqualified on account of mental deficiency or defectiveness, and on that account an "idiot" or "insane."

---

*The annual address before The American Medico-Psychological Association, at SanAntonio, Texas, April, 1905.

All kinds and grades of mental abnormalities are, however, psychoses, and are every-day matters of observation, concern and care on the part of medical men.

Mentality is the most important of all studies. It is a matter of vital importance to the individual, because his welfare, safety and degree of success depend upon the grade of its efficiency within himself, and no general subject concerns the public more. The average level of the mentalities of its citizenship determines the standing of the community, the race, or the nation.

For these reasons, psychology, which is the study or the science of normal mentality, is rapidly attracting more and more popular and scientific attention, and psychiatry, which is the study or science of abnormal mentality, is assuming more and more scientific aspect.

As our pathology is the study of deficient, defective or disturbed physiology, so our psychiatry is the study of deficient, defective or disturbed psychology.

If I approach the subject of normal and abnormal mentality from a rather new direction, in this august presence of psychologists and psychiatrists from over a whole continent, I do it with extreme diffidence. My suggestions are, of course, only tentative, even by myself subject to ready change as they are altered by further instruction and information.

Let me at this point express my appreciation of the high compliment included in the appointment of your Committee of Arrangements for me to make the annual address at this meeting of the Association. The very prominence of the position embarrasses me, and I shall have to bespeak your kind indulgence, for I am simply an inconspicuous member from among you.

### TRIPARTITE MENTALITY
### OR
### THREE NATURAL DIVISIONS OF MENTALITY.

In a broad sense, mentality may be said to be a property exhibited by all living things. For this reason, it can be observed, classed and studied throughout the whole biologic world.

In scientific philosophy the usual method is to accurately observe, grade and classify phenomena everywhere. Then, when sufficient data have been accumulated, to use the more familiar and simpler to explain the unusual and more complex of the same class. This has been very generally the rule in philosophizing about the inanimate world. It has, however, not so generally been the rule in philosophizing about the animate world; of late, however, this method has been more generally adopted. We are beginning to use simpler mental phenomena to explain the more complex.

All living things exhibit certain common characteristics, which we look for to determine whether they are alive. First among these may be mentioned sentiency, with which we observe the living thing recognizes impressions made on it from its environment; secondly, accompanying this faculty, as a part of it, the living thing receives into its sentient structures information of the impressions made on it; thirdly, we observe the living thing emits motions in response to the information it has received; fourthly, we observe that by an intermediate act of this same sentient faculty the living thing has adjusted its emitted acts to its received ones, so as to conserve its welfare and safety.

Viewed in this way, wherever exhibited, mentality shows itself as tripartite in character. It is readily divided into three departments; it receives, it adjusts and it emits; or, to use more complex psychologic terms, it learns, it reasons, it executes.

Even the highest reaches of mental effort, I think, can be classed with considerable efficiency into three such departments. The receiving department, for instance, can include all the mental acts of appreciating, understanding, comprehending, acquiring, learning; the emitting department can include all the intentions, conclusions, wishes, purposes, decisions—the results of the adjustments and ratiocinations made upon and out of the acquisitions; the propositionizing of sentences lies in this department, and the election or inhibition of the results of the reasoning department presented for emission—most of them, probably, are turned back for further preparation. The inhibitory part of mentality constitutes, on the emissive side, a large and important part of its work. Intermediate between the learning and the executing departments, lies the department of reason or adjustment, made efficient in its complexity by the faculty of recollecting or remembering previous mental acts—probably by the ability to perform them again. This department can dissociate its acts, largely sometimes, from the immediate receptions and emissions in the "flights" of imagination and abstract ideation.

It is impossible here to follow into detail this line of generalization of mentality into its three departments. I simply draw attention to it, hoping it may prove of value because of its apparent naturalness.

Every living thing which is organized is composed of innumerable living cells. The cell is the unit of organized life. It is a living entity in itself. Like the whole animal, it has to work for its own support, while it has functional work in its environment. For these purposes it has its receiving, its adjusting and its emitting structures and activities.

Similar cells are aggregated and associated together in composing the structures of an organ of the body, and each organ, in its aggregate capacity, has its receiving, adjusting and emitting faculties; not only in accomplishing its special function, but in adjusting its acts to those of other organs.

Air (or oxygen), water, warmth and food (not to mention light), may be classed as "the necessaries of life." Every living cell within the body has to have these "necessaries" in order to live. To obtain from outside and convey internally to the cells these necessaries is the first object of effort on the part of every living thing. All living things are organized, principally, for this object, and there are all kinds and grades of complexity in the organic construction of different living things for this purpose.

Air and water, while most highly necessitous, are so abundant and easily obtained as not to be so much objects of effort and concern as the other two. To obtain warmth, in the shape of shelter, clothing, fuel and heat-making food, is more a matter of concern and effort. Food for cell-heating and to repair cell wastes, because it is the scarcest and most difficult to obtain, is the highest priced and the object of most concern and effort.

"To work for a living" is a common expression, illustrating the fact that man, primarily, has to work for "the necessaries" in order to live. "To accumulate wealth and property" principally means to secure an abundant provision of them for present and for future use.

To go no further than to take "the necessaries of life" as objects of activity of the man without himself and within himself, it is easy to divide the body into two general departments of organs and functions. The one department relates to the obtaining of the necessaries from the environment, and the other to their preparation and distribution to the cells everywhere within. Man is highly organized for these two objects.

Of course, other objects of external effort present themselves as the man grows older, until the summation of all eventually becomes very complex. To improve himself by improving his mental abilities and by the acquisition of knowledge, as well as to improve his environment by increasing the general store of knowledge are objects of effort. The improvement of his environment by the removal of offensive, dangerous and troublesome agents, is another object, as well as cultivating and encouraging things that contribute to his welfare and happiness. The improvement of his environment by the improvement of his fellow-men, physically, mentally and morally, is another. These, with other matters, all make exceedingly complex the department which relates to the external world.

The internal department principally relates to the preparation and distribution of the "necessaries of life," though, in addition, there is the removal of waste material and of toxins from the system, and, in the female particularly, the caring for the coalesced embryo of the line-of-descent. Other objects of internal work could be mentioned.

The division of the body into two general departments is well illustrated in sleep, when the highest and most important department which relates to the environment is suspended for repair. The high-brain, the cortex, with its lines of afferent action, from the organs of sense, and its efferent lines, which put in motion the "voluntary muscles," all are dormant at that time, while the internal department continues in acting, distributing "the necessaries of life" and removing waste.

The same division is aptly shown in the administration of an anesthetic. The chemic action of the drug, distributed generally in the circulation, affects first the most delicate structures of the high brain and suspends their action. The consciously-sentient cortex, with its lines of reception and of emission, is stopped in function, while the less sentient sub-conscious, low-grade, nerve-center structures, relating to internal action, are not affected so much, and continue to act. The administration of the anesthetic can ordinarily be safely held at that point. If, however, it is carried further, so as to paralyze the adjusting centers that equilibrate and control the actions of the internal organ, the man is killed.

About a hundred years ago Sir Charles Bell discovered, or more distinctly determined, the functions of "the sensory and motor roots of the spinal cord." This was not a very important discovery in itself, but it soon became so, because, like the discovery of Columbus of a small island in the West Indies, a whole continent of contingent discoveries have followed it.

The posterior columns of the cord have since been found to carry ascending, aerent, receptive, informing, actions to the cortex, and to go posteriorly into it, while the anterior columns carry descending, efficient mandatory actions to the muscles. This has been followed also by a mapping out of a large portion of the posterior cortex into receptive tracts, while anteriorly emissive actions are known to leave it. Decussating, connecting fibres, all along in the cord, anterio-posteriorly and laterally, make its tracts or centers of nerve cells into adjusting levels for controlling the actions of parts below them. Such adjusting functions are shown to act with more and more scope and efficiency as they ascend in the cord, in the pons, in the lower and in the higher brain.

If we begin at the highest center, the cerebral cortex, and study down through the nervous system we find a descending gradation of sentient structures all with their receiving, their adjusting and their emitting functions.

The high brain, which is engaged in the highest and most complex work of adjusting to the environment, is immensely complex in structure, commensurate with its immensely complex work, and has, in part, been mapped over as performing its receiving work anteriorly; while over-riding, pervading and subtending all are the adjusting functions for preparing out of the receptions and acquisitions the emissive conclusions, purposes, wishes and designs.

From the sense organs, of seeing, hearing, smelling, tasting, and from the general sentiency of the whole body, along afferent nerve fibres, comes information, posteriorly, into the cortex; while anteriorly, along efferent nerve fibres, go mandates putting into execution the results of the adjusting ratiocination. The transition of an act through the brain from rear to front may be "as quick as thought" or as deliberate as a lifetime.

At the furthest extreme from the cortex, in the periphery, a small vaso-motor ganglion, with the simplest function, for instance, of regulating the caliber of a capillary, receives, adjusts and emits in its action, and so do the ganglionic centers of the sympathetic system, harmonizing and regulating some of the actions of the organs of the internal department; tracts in the cord, in the pons and in the base of the brain act similarly.

The man is an entirety from head to foot. There is no distinct line of separation between the departments for external and internal adjustments either in the nervous system or in the other organs. Disturbance or shock in the one is promptly carried into the other. Suddenly or slowly, as the case may be, one department is impaired by disturbing action in the other. Still, there is a separateness.

Pain may be said to be occasioned by the transition of disintegrating cellular action, going on in the periphery along afferent fibres to the cortex. It may not stop at, but come through centers of a lower grade, sense of whose actions does not usually reach the cortex, for they are carried on with sub-conscious sentiency.

I think it is interesting to study mental operations of the highest order with this tripartite classification in mind.

For instance, from infancy to adult life, mental ability in each of the departments—of learning, of reasoning and of executing——gradually improves.

To acquire information or knowledge [illegible] necessarily the first object of mental effort, requiring steady or continuous attention. For this reason the acquiring faculties start first in the growing child, and as he grows older precede in their gradual development, the reasoning and the executive. In our educational methods we take advantage of the fact that early life is the best acquiring age. We can do our teaching and training best during that period. Apparently the acquiring faculties in man develop faster and reach maturity soonest. He does the best part of his learning and his training in the first third of his average lifetime. The second part of his growing mentality, his reasoning faculty, his "best judgment," does not reach maturity until middle life, and the third part, his emissive or his executive department, matures after his learning and his reasoning. Mentality naturally develops and matures in this order in life, and, I think, naturally declines in the senescence of old age in the same order. It is hard to teach new ideas or new methods to an old man, but in lines in which he is practiced, experienced and long-ago taught, his judgment and the output of his emitted opinions are often full of "wisdom."

Of the scholars in a school a goodly proportion, probably a third, generally exhibit excellent learning abilities; in middle life, when reasoning abilities mature, a much smaller number of them exhibit tenacity of purpose and good executive ability. The average man falls behind in excellence in this order as he takes part in competitive life. The fully rounded, excellent man exhibits a high order of abilities in all three departments. He starts out as a good scholar, and has good judgment and his emissive work afterwards show themselves to be of a high grade. Ability to be excellent in the second and third departments as well as the first constitutes the successful man. The simply erudite scholar is excellent in acquiring abilities, but fails in judgment and execution.

The teacher reaches principally the acquiring department of his scholar. The mental gymnastics he makes the scholar do principally improve his learning abilities. The reasoning department and the executive are largely out of the teacher's reach, and mature later. They are born into the man, not schooled into him. If not degenerated in him, they are ancestral, family or race traits largely. "Common sense." which means ability all through life to learn, reason and execute in a practical, profitable way, is especially born into the man.

I have ventured to speculate this much in the line of the highest order of mentality to show how far we can carry this tripartite division. In this direction I believe will lie the coming field of scientific investigation and generalization.

As psychologists, as we know more accurately the tracts in the cortex, we will be more able to observe and classify normal mental activities and abilities, and as psychiatrists this same natural method of making observations and conclusions will the better enable us to determine the abnormal deficiency or defect—whether of a local or of a general character.

